# An endometrial receptivity scoring system evaluated by ultrasonography in patients undergoing frozen–thawed embryo transfer: a prospective cohort study

**DOI:** 10.3389/fmed.2024.1354363

**Published:** 2024-03-21

**Authors:** Yan Ouyang, Yangqin Peng, Yuyao Mao, Mingxiang Zheng, Fei Gong, Yuan Li, Xihong Li

**Affiliations:** ^1^Reproductive and Genetic Hospital of CITIC-Xiangya, Changsha, China; ^2^Clinical Research Centre for Reproduction and Genetics in Hunan Province, Changsha, China; ^3^School of Medicine, Hunan Normal University, Changsha, China; ^4^NHC Key Laboratory of Human Stem Cell and Reproductive Engineering, School of Basic Medical Sciences, Central South University, Changsha, China

**Keywords:** endometrial receptivity, three-dimensional ultrasound, ultrasonography, prospective, scoring system, noninvasive

## Abstract

**Introduction:**

Ultrasound has become a routine method for endometrial receptivity (ER) evaluation. However, there is controversy over the independent evaluation values of various ultrasound indicators. Some researchers have designed multi-indicator prediction systems, but their prediction values are uneven. To further our understanding of ER, we conducted this prospective cohort study to estimate ER noninvasively and effectively.

**Methods:**

Women who underwent the first frozen–thawed embryo transfer (FET) cycle from April 2019 to July 2021 were included in the study. On the day of transfer, transvaginal three-dimensional ultrasound examination was performed to evaluate ER, including endometrial thickness, morphology, volume, movement, blood flow and flow index. The clinical pregnancy rate was the primary outcome. Based on whether clinical pregnancy was achieved, enrolled patients were divided into pregnant and nonpregnant groups.

**Results:**

This study analyzed 197 FET patients (139 pregnancies in total, 70.5%). The protective factors for clinical pregnancy included primary infertility [adjusted odds ratio (aOR), 1.98; 95% confidence interval (CI), 1.01–3.882; *p* = 0.047] and more frequent endometrial peristalsis (aOR, 1.33; 95% CI, 1.028–1.722; *p* = 0.03). Scores of 1–2 were assigned according to the relationship between different ultrasound indicators and the clinical pregnancy rate (CPR). The ER score of the patient was the sum of the scores of the 6 items. The ER score of the pregnant group was significantly higher than that of the nonpregnant group (7.40 ± 1.73 vs. 6.33 ± 1.99, *p* = 0.001). The CPR increased with an increasing ER score. The CPR in the ER < 6 group was significantly lower than that in the ER >6 group (45.5% vs. 75.6%, *p* = 0.001).

**Conclusion:**

A noninvasive ultrasound scoring system for ER was proposed. This system may provide a non-invasive guidance perspective, in conjunction with invasive assessments currently used in clinical practice, to achieve more effective embryo transfer.

## Introduction

The capacity of the endometrium to allow embryo implantation and subsequent development is known as endometrial receptivity (ER) (1). Embryo quality and ER are two key factors affecting embryo implantation during *in vitro* fertilization-embryo transfer (IVF-ET). The endometrium plays an important role in the process of embryo implantation, especially when the embryo is of good quality. The most accurate way to evaluate uterine ER is endometrial biopsy and histopathological examination (2). However, due to the invasive nature of the examination, its clinical application is limited. In recent years, the ultrasound technique has become a routine approach for the evaluation of ER due to its advantages of safety, noninvasiveness, simple operation and strong repeatability.

There is controversy over the independent evaluation values of various ultrasound indicators. It has been shown that the likelihood of pregnancy increases after a certain endometrial thickness threshold is reached (3). An endometrium that is too thin or too thick has been reported to be unfavorable for embryo implantation (4, 5). Moreover, some studies have reported that endometrial thickness is not a good predictor of pregnancy outcomes, with limited predictive value (6, 7).

Some studies indicate that the endometrial morphology on the human chorionic gonadotropin (HCG) injection day can predict pregnancy outcomes and that the appearance of the triple line sign indicates a higher pregnancy rate (8). However, a meta-analysis showed that there was no significant difference in endometrial morphology between the pregnant group and the nonpregnant group on the day of HCG injection and transfer (2). This meta-analysis also reported that endometrial volume (EV) was not significantly associated with pregnancy in fresh embryo transfer cycles. However, some other studies have suggested a correlation between EV and pregnancy (9–11).

A meta-analysis showed that the frequency of endometrial peristalsis on the day of transfer was negatively correlated with the pregnancy rate (12). Chung et al. (13) found that there was no significant difference in the frequency of endometrial peristalsis 5 min before transfer between the pregnant group and the nonpregnant group, but the frequency of endometrial peristalsis 5 min after transfer decreased in the pregnant group, suggesting that the frequency of endometrial peristalsis 5 min after transfer may be an important predictor of IVF outcomes.

The endometrium is the implantation site of embryos. Most studies (14, 15) have reported that the pregnancy outcomes of women with good endometrial and subendometrial blood flow perfusion are better, but the most significant predictor has not been determined. Early studies reported that uterine artery blood flow parameters might be reliable indicators for evaluating ER (16, 17). However, most studies now report that uterine artery blood flow parameters and the pregnancy rate are not directly related (18, 19).

The differences in the results of a single ultrasound index may be attributed to discrepancies in the study design, study populations, ovulation induction schemes, insemination methods, measurement time points, etc. Because the predictive value of a single index is difficult to determine, some researchers have designed multi-index comprehensive evaluation and prediction systems. To date, there have been 6 studies of comprehensive scoring systems (20–25), but the measurement time, inclusion indices, assigned scores and prediction value are uneven.

To further our understanding of ER, we conducted this prospective cohort study with patients undergoing a natural frozen–thawed embryo transfer (FET) cycle after the first stimulated IVF treatment. Furthermore, we built a simple scoring system using ultrasound indicators on the day of transfer to assist in clinically estimating ER in a noninvasive and effective manner.

## Methods

### Study design and participants

A total of 197 infertile women who underwent FET cycles at the Reproductive and Genetic Hospital of CITIC-Xiangya from April 2019 to July 2021 were included. All participants signed written informed consent. This study was approved by the Ethics Committee of the Reproductive and Genetic Hospital of CITIC-Xiangya (date of approval: 11 September 2019; reference number: LL-SC-2019-023; Changsha, China).

The inclusion criteria were as follows: (1) patients who received FET after their first stimulated IVF treatment and had no history of assisted reproduction in other hospitals; (2) patients who underwent a natural FET cycle; (3) patients aged 20–35 years old; (4) patients with a body mass index of 18–24 kg/m^2^; (5) patients who had at least 1 high-quality embryo; (6) patients who underwent ultrasonic examination in our hospital, with clear image quality; (7) patients with correct data and complete records; and (8) patients with no history of uterine cavity surgery. If endometrial polyps were found, they would be removed by routine hysteroscopy prior to embryo transfer.

**Figure 1:**
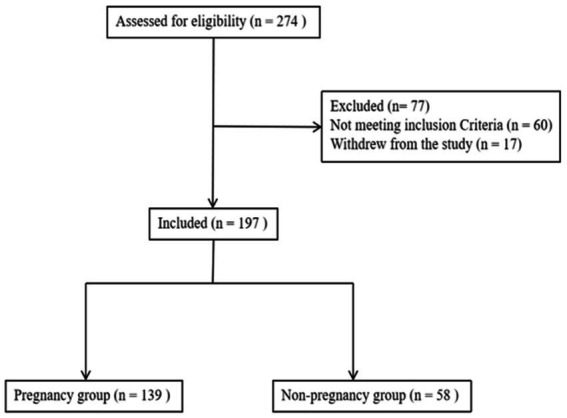
Flowchart of the included patients. A total of 274 patients were assessed for eligibility, after excluding 77 patients, 197 patients were finally included for analysis.

The exclusion criteria were as follows: (1) patients with endometriosis, adenomyosis, or adenomyoma; (2) patients with intrauterine adhesion or endometritis; (3) patients with congenital uterine anatomy malformations; (4) patients with untreated hydrosalpinx; and (5) patients with endometrial cavity fluid caused by a cesarean section incision and fluid diameter ≥ 2 mm. If a patient met one of these items, the patient was excluded. The flow diagram for patient inclusion is shown in Figure 1.

### *In vitro* fertilization procedure

All included patients underwent controlled ovarian hyperstimulation (using follicle-stimulating hormone or human menopausal gonadotropin) and HCG injection. Fertilization was achieved using either standard IVF or intracytoplasmic sperm injection (ICSI), depending on the cause of infertility. Frozen embryos were transferred during the natural cycle for all included women. According to the recommendations of the freezing and thawing kit (Vitrolife Sweden AB), 1,2-propanediol and sucrose were used as cryoprotectants. For natural cycles, endometrial thickness and follicle diameter were monitored by transvaginal sonography (TVS) from days 10–12 of the menstrual cycle. Thawed cleavage-stage embryos were transferred on the 3rd day after ovulation, and blastocysts were transferred on the 5th day after ovulation.

All patients underwent high-quality embryo transfer. Embryo morphology was scored according to the Alpha Scientists in Reproductive Medicine and ESHRE Special Interest Group of Embryology (ASEBIR) consensus (26). An embryo with at least 7 blastomeres, a fragmentation rate ≤ 10%, uniform and consistent blastomeres, no vacuoles, normal zona pellucida and blastocysts ≥3BB grade were defined as high-quality embryos. Up to two embryos could be transferred.

### Ultrasound measurement

Ultrasound scans were performed in the morning on the embryo transfer day to evaluate ER in the included patients. All ultrasound parameters, including endometrial thickness, morphology, volume, movement and blood perfusion, were measured by the same doctor (Dr. Li) using the same ultrasound machine (GE VOLUSON E8, General Electric Tech Co., Ltd., New York, United States) equipped with a 5–9 MHz transvaginal three-dimensional (3D) probe.

### Two-dimensional grayscale ultrasound mode

#### Endometrial thickness and morphology

**Figure 2:**
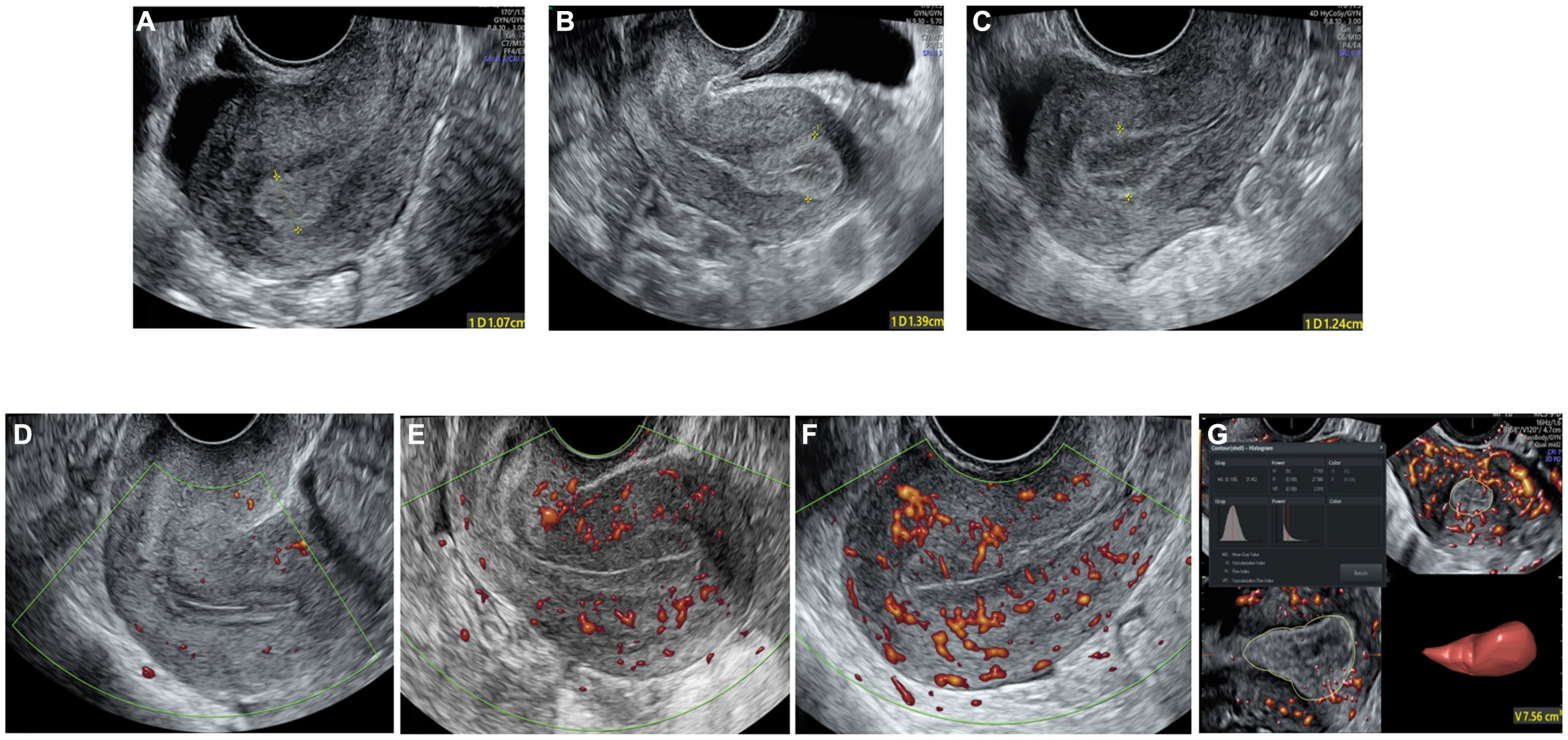
The two-dimensional grayscale image shows the endometrial morphology. **(A)** Type A endometrial pattern; **(B)** type B endometrial pattern; **(C)** type C endometrial pattern. Power Doppler shows the blood flow pattern of the endometrium. **(D)** Blood flow pattern I; **(E)** blood flow pattern II; **(F)** blood flow pattern III. **(G)** Three-dimensional image showing the vascularization parameters and endometrial volume.

The maximum diameter of the endometrium was measured in the longitudinal plane. The Gonen classification criteria (27), in which the endometrial pattern is determined by comparing the echo of the endometrium to that of the adjacent myometrium, were adopted to evaluate endometrial morphology as follows: Type A: an entirely homogeneous, hyperechogenic endometrium with increased reflectivity and no visualized central echogenic lines; Type B: an endometrium with the same reflectivity as the surrounding myometrium, with a nonprominent or absent central echogenic line; and Type C: a “triple-line” endometrium, consisting of prominent outer and central hyperechogenic lines and inner hypoechogenic or black regions (Figures 2A–C).

#### Endometrial peristalsis

The movement of the endometrium was observed and recorded within 3 min to observe whether there was endometrial peristalsis and its direction of movement. Ijland et al. (28) classified endometrial peristalsis into 5 types: (1) positive wave: the peristaltic wave from the cervix to the fundus; (2) negative wave: the peristaltic wave from the fundus to the cervix; (3) static wave: the endometrium is in a static state; (4) bidirectional wave: the endometrium at the fundus and the cervix contract simultaneously; and (5) random wave: an irregular movement type with an uncertain direction or multiple starting points. Take the positive wave as an example, when the wave starting from the cervix reaches the fundus of the uterus, there will be an opposite wave from the fundus of the uterus back to the cervix, and we calculate these two back and forth waves as one wave.

### Blood flow distribution

#### Endometrial blood flow

Blood flow distribution was observed in the longitudinal plane of the uterus. Endometrial blood perfusion was classified as follows based on the Applebaum classification standard (20): I: blood vessels pass through the outer hypoechoic area and surround the endometrium but do not enter the hyperechoic endometrial margin; II: blood vessels penetrate the hyperechogenic outer margin of the endometrium but do not enter the hypoechogenic inner area; and III: blood vessels enter the hypoechoic intraendometrial area (Figures 2D–F).

### 3D ultrasound examination

#### Endometrial volume and vascularization index

The ultrasound machine was switched to the power Doppler 3D mode. The sector of interest was adjusted to cover the endometrial cavity in the longitudinal plane, and the sweep angle was set to 90 degrees to ensure that the whole uterine volume was scanned. The 3D volume was acquired while keeping the transvaginal probe still, and virtual organ computer aided analysis (VOCAL) software was used for analysis. The endometrium was outlined, and then the EV and endometrial vascularization index (VI), vascularization flow index (VFI) and flow index (FI) were obtained (Figure 2G) (29, 30).

#### Outcome measures

The primary outcome of this study was the clinical pregnancy rate (CPR). Serum human HCG levels were measured 14 days (12 days after blastocyst transfer) after transfer, and TVS scans were performed 4 weeks after transfer to examine the pregnancy location and viability. Ultrasound visualization of an intrauterine pregnancy was considered a clinical pregnancy. Based on whether clinical pregnancy was achieved, enrolled patients were divided into pregnant and nonpregnant groups.

### Statistical analysis

The Kolmogorov–Smirnov test was used to analyze the demographic distribution of patients. Continuous variables are represented herein by the mean ± standard deviation (SD). Categorical variables are expressed as frequencies and percentages. Mann–Whitney U tests or Student’s *t*-tests were used to assess continuous variables, and chi-square tests or Fisher’s exact tests were used to assess differences in categorical variables between the pregnant group and the nonpregnant group. Variables with *p* values less than 0.05 in univariate logistic regression analysis were included in multivariate logistic regression analysis to identify independent predictors of clinical pregnancy with the forward likelihood ratio stepwise selection method; odds ratios (ORs) and 95% confidence intervals (CIs) were calculated. A receiver operating characteristic (ROC) curve was used to discriminate the predictive values of each independent predictor separately or jointly in the multivariate logistic regression model for the clinical outcome (pregnant group vs. nonpregnant group) and to evaluate the predictive performance of the total receptivity scores of six endometrial ultrasound markers for clinical pregnancy in the training sample and the verification samples separately, and areas under the curve (AUCs) and 95% CIs were calculated. SPSS 25.0 software (IBM, Armonk, NY, United States) was used for all statistical analyses, and a difference of *p* < 0.05 was considered statistically significant.

## Results

### Patient characteristics and ultrasound parameters

A total of 197 patients who underwent FET were included. The overall CPR was 70.5% (139/197). The univariate analysis of basic, clinical and endometrial ultrasound features of the two groups is displayed in Table 1. The proportion of women with secondary infertility in the pregnant group was significantly lower than that in the nonpregnant group (54.0% vs. 70.7%, *p* = 0.030). There were no statistically significant differences in female age, duration of infertility, cause of infertility, body mass index, antral follicle count, basal hormonal levels, number of embryos transferred, or proportion of the stages of embryos transferred between the two groups (*p* > 0.05).

**Table 1:** Comparisons of the characteristics and ultrasound parameters between the two groups.

Parameters	Nonpregnant group (*n* = 58)	Pregnant group (*n* = 139)	*p*-value
Age (years)^a^	30.1 ± 3.5	30.0 ± 3.3	0.708
Duration of infertility (years)^a^	4.1 ± 2.9	3.8 ± 2.2	0.698
BMI (kg/m^2^)^a^	21.2 ± 1.7	21.4 ± 1.7	0.519
Type of infertility^b^			0.030
Primary	17(29.3%)	64(46.0%)	
Secondary	41(70.7%)	75(54.0%)	
Cause of infertility, n (%)^c^			0.345
Unexplained	0(0%)	3(2.2%)	
Male factor	3(5.2%)	8(5.8%)	
Female factor	31(53.4%)	61(43.9%)	
Male and female factors	24(41.4%)	67(48.2%)	
AFC^a^	21.3 ± 8.7	23.3 ± 13.8	0.742
Basal hormonal levels^a^			
FSH (mIU/mL)^a^	5.8 ± 1.2	5.8 ± 1.7	0.357
LH (mIU/mL)^a^	3.6 ± 1.3	4.1 ± 4.2	0.799
E2 (pg/mL)^a^	37.0 ± 15.6	42.2 ± 28.6	0.724
PRL (ng/mL)^a^	18.6 ± 20.6	20.4 ± 17.3	0.095
P (ng/mL)^a^	1.0 ± 2.6	0.8 ± 2.7	0.505
AMH (ng/mL)^a^	5.9 ± 3.9	5.7 ± 4.5	0.323
Number of embryos transferred^b^	1.5 ± 0.5	1.7 ± 0.5	0.139
1	27(46.6%)	49(35.3%)	
2	31(53.4%)	90(64.7%)	
Stage of embryo transferred^b^			0.994
Cleavage stage embryo	25(43.1%)	60(43.2%)	
Blastocyst	33(56.9%)	79(56.8%)	
Endometrial morphology classification^c^			0.448
Type A	7(12.1%)	12(8.6%)	
Type B	50(86.2%)	120(86.3%)	
Type C	1(1.7%)	7(5%)	
Endometrial blood flow classification^c^			0.563
I	12(20.7%)	24(17.3%)	
II	45(77.6%)	109(78.4%)	
III	1(1.7%)	6(4.3%)	
Endometrial thickness (mm)^d^	11.8 ± 2.6	11.9 ± 2.2	0.917
Endometrial 3D indicators			
Volume (mL)^a^	3.9 ± 1.5	4.1 ± 1.7	0.371
VI^a^	0.5 ± 0.7	0.5 ± 0.6	0.553
FI^a^	25.3 ± 8.0	27.6 ± 6.8	0.016
VFI^a^	0.2 ± 0.3	0.2 ± 0.6	0.313
Frequency of endometrial peristalsis (times/min)^a^	1.0 ± 1.2	1.5 ± 1.4	0.021

^a^Mann–Whitney U test. ^b^Chi-squared test. ^c^Fisher’s exact-probability test. ^d^Two-sample *t*-test. BMI, body mass index; AFC, antral follicle count; FSH, follicle-stimulating hormone; LH, luteinizing hormone; E2, estradiol; PRL, prolactin; P, progesterone; AMH, anti-Mullerian hormone; 3D, three-dimensional; VI, vascularization index; FI, flow index; VFI, vascularization flow index.

**Figure 3:**
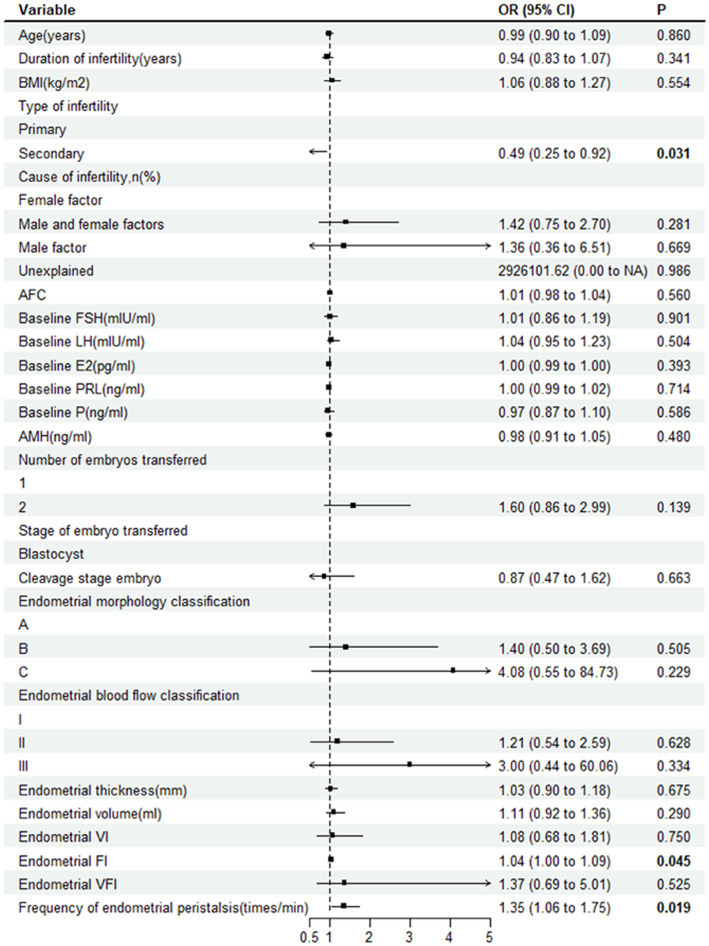
Forest plot showing the relationship between various indicators and clinical pregnancy. OR, odds ratio; BMI, body mass index; AFC, antral follicle count; FSH, follicle-stimulating hormone; E2, estradiol; PRL, prolactin; P, progesterone; AMH, anti-Mullerian hormone; VI, vascularization index; FI, flow index; VFI, vascularization flow index.

When comparing the ultrasound parameters of ER between the two groups, the FI (27.6 ± 6.8 vs. 25.3 ± 8.0, *p* = 0.016) and frequency of endometrial peristalsis (1.5 ± 1.4 vs. 1.0 ± 1.2 times/min, *p* = 0.021) in the pregnant group were significantly higher than those in the nonpregnant group. Other indicators, such as the endometrial morphology classification, endometrial blood flow classification, endometrial thickness, EV, VI, and VFI, were not significantly different between the two groups (*p* > 0.05). The forest plot of univariate logistic regression analysis shows the relationship between various indicators and clinical pregnancy. The type of infertility (OR 0.49; 95% CI, 0.25–0.92; *p* = 0.031), endometrial FI (OR 1.04; 95% CI, 1.00–1.09; *p* = 0.045) and frequency of endometrial peristalsis (OR 1.35; 95% CI, 1.06–1.75; *p* = 0.019) were found to be significantly correlated with clinical pregnancy (Figure 3).

### Multivariate logistic regression

The factors that were significant after univariate logistic regression analysis were included in the multivariate forward likelihood ratio stepwise selection logistic regression analysis to obtain the ORs and 95% CIs of the independent risk factors contributing to clinical pregnancy. The results showed that the type of infertility and frequency of endometrial peristalsis were independent predictors of clinical pregnancy after FET. Protective factors for clinical pregnancy in the FET cycle include primary infertility (aOR, 1.98; 95% CI, 1.01–3.882; *p* = 0.047) and more frequent endometrial peristalsis (aOR, 1.33; 95% CI, 1.028–1.722; *p* = 0.03).

The ROC curve analysis of the type of infertility and frequency of endometrial peristalsis showed that the AUCs were 0.584 (95% CI: 0.498–0.670) and 0.602 (95% CI: 0.517–0.686), respectively. Furthermore, ROC curve analysis was used to assess the predictive capacity of the combination of the type of infertility and frequency of endometrial peristalsis, and the AUC was 0.633 (95% CI: 0.552–0.714), which was higher than the AUC determined solely by the aforementioned 2 indicators separately.

### ER scoring system and ROC curve analyses

To comprehensively reflect ER, we assessed 6 ultrasound markers, including the endometrial morphology classification, blood flow classification, thickness, volume, frequency of peristalsis and FI, to establish an ER scoring system (Table 2).

**Table 2:** Endometrial receptivity scoring system and the clinical pregnancy rate.

Ultrasound index	Score			Clinical pregnancy rate (n/N)
	0	1	2	
Endometrial morphology				
Type A	0			63.2% (12/19)
Type B		1		70.6% (120/170)
Type C			2	87.5% (7/8)
Endometrial blood flow				
I	0			66.7% (24/36)
II		1		70.8% (109/154)
III			2	85.7% (6/7)
Endometrial thickness*				
<9 mm	0			47.1% (8/17)
9–14 mm			2	73.9% (105/142)
>14 mm		1		68.4% (26/38)
Endometrial volume				
≤2 mL	0			41.7% (5/12)
>2 mL			2	72.4% (134/185)
Endometrial flow index (FI)				
<26	0			60.7% (37/61)
26–31		1		69.5% (57/82)
>31			2	83.3% (45/54)
Frequency of endometrial peristalsis				
<1	0			65.1% (56/86)
1–2		1		69.8% (44/63)
>2			2	81.3% (39/48)

FI, flow index. * When the endometrium is between 9 and 14 mm, the score is 2 instead of 1.

First, the patients were divided into three groups according to the endometrial morphology classification (groups I, II and III), with pregnancy rates of 66.7, 70.8, and 85.7% and endometrial receptivity scores of 0, 1, and 2, respectively. Similarly, the endometrial blood flow classification was divided into A, B and C, with pregnancy rates of 63.2, 70.6, and 87.5% and receptivity scores of 0, 1, and 2, respectively. The endometrial thickness was divided into three groups with cutoffs of 9 mm and 14 mm (<9, 9–14, and >14 mm), with pregnancy rates of 47.1, 73.9, and 68.4% and receptivity scores of 0, 2, and 1, respectively. The CPR was higher in patients whose EV was >2 mL; therefore, these patients received a score of 2, while patients whose EV was ≤2 mL received a score of 0 (72.4% vs. 41.7%, respectively). Likewise, the endometrial FI was divided into 3 groups with cutoffs of 26 and 31 (< 26, 26–31 and > 31), with pregnancy rates of 60.7, 69.5, and 83.3% and receptivity scores of 0, 1, and 2, respectively. The patients with endometrial peristalsis occurring <1 time/min were assigned a score of 0, those with endometrial peristalsis occurring 1–2 times/min were assigned a score of 1, and those with endometrial peristalsis occurring >2 times/min were assigned a score of 2 (pregnancy rates: 65.1, 69.8, and 81.3%, respectively).

**Figure 4:**
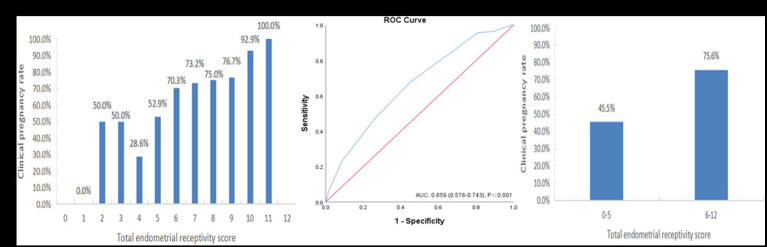
The performance of the endometrial receptivity scoring system in the training sample. **(A)** Clinical pregnancy rate associated with each total endometrial receptivity score. **(B)** ROC curve analysis for the scoring system. **(C)** Clinical pregnancy rate in the groups with total endometrial receptivity scores <6 and ≥ 6. ROC, receiver operating characteristic; AUC, area under the curve.

The summed receptivity scores of the 6 ultrasound markers resulted in a total score ranging from 0 to 12, and the total receptivity scores in the pregnant group and nonpregnant groups were 7.40 ± 1.73 and 6.33 ± 1.99, respectively, which were significantly different (*p* = 0.001). The pregnancy rates of each total score group are shown in Figure 4A. The CPR increased with increasing ER score, and the CPR improved markedly when the score reached 6.

Figure 4B depicts the ROC curve of the ER scoring system (AUC = 0.659, 95% CI: 0.576–0.743), and the cutoff value was point 6, with a sensitivity of 73.9% and a specificity of 64.7%. For further analysis, we divided the total endometrial receptivity score into two groups, with a cutoff value of 6. Figure 4C shows that the total ER score increased from the <6 group to the ≥6 group, indicating a significant improvement in clinical pregnancy rates (45.5% vs. 75.6%, *p* = 0.001).

### Verification of the scoring system

**Figure 5:**
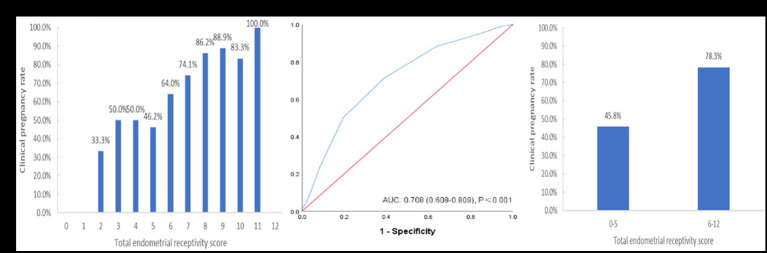
The performance of the endometrial receptivity scoring system in the verification sample. **(A)** Clinical pregnancy rate associated with each total endometrial receptivity score. **(B)** ROC curve analysis for the scoring system. **(C)** Clinical pregnancy rate in the groups with total endometrial receptivity scores <6 and ≥ 6. ROC, receiver operating characteristic; AUC, area under the curve.

After building the scoring system, we included an additional 130 patients from August to June 2023 with the same inclusion criteria of this system for validation. The results also showed that the CPR increased with an increasing ER score (Figure 5A). The ROC curve analysis showed that the AUC of this verification sample was 0.708 (95% CI: 0.608–0.809) (Figure 5B). The CPR of the group with a total ER score of ≥6 was also significantly improved compared to that of the group with a total ER score of <6 (45.8% vs. 78.3%, *p* = 0.001) (Figure 5C).

## Discussion

In this prospective study, an ultrasound scoring system for ER was proposed, and its evaluation value and ability were verified. With this system, a patient’s ER can be evaluated noninvasively, efficiently and accurately, which is helpful to indicate whether embryo transfer is suitable and to provide patients with corresponding treatment advice.

Primary infertility and peristaltic waves were shown to be protective factors against clinical pregnancy during FET cycles in this study. We speculate that primary infertility patients are less likely to have destroyed endometria and more likely to have better receptivity. However, the relationship between peristaltic waves and the ER has been controversial in previous studies. Some studies (28, 31) have reported that endometrial peristalsis should be static during transfer, while Chung et al. (13) believed that there is no difference in endometrial peristalsis 5 min before transfer and that the reduction in peristalsis 5 min after transfer plays a positive role in pregnancy. However, our study showed that the frequency of peristalsis on the transfer day was greater in the pregnant group than in the nonpregnant group.

First, the reason for the difference may be that the measurement time was different. All of our measurements were taken on the morning of embryo transfer (transfer in our hospital is usually arranged in the afternoon). In fact, we do not know whether a change in endometrial peristalsis occurs in the short period (such as 5 min) before and after transfer. It cannot be ruled out that the endometrium tends to be static a few minutes before and after transfer, which is what we need to further investigate in the future. Second, it is necessary to further explore whether endometrial peristalsis in natural menstrual cycles and stimulation cycles is different under the effect of different hormone levels. Third, the measurement is subjective. For example, in the literature, it is only mentioned that the peristalsis of the endometrium from the cervix to the fundus is a positive wave (28), but in actual observation, peristalsis from the cervix to the fundus is generally followed by an echo. Our hospital calculated a one-loop wave at the beginning of the cervix as a positive wave, but this does not rule out that some studies have calculated the one-loop wave as a positive or negative wave. However, at present, there is no further in-depth discussion on this issue in the various studies. We should also note that the ROC areas of primary infertility, peristaltic waves and the combination of these two are not very superior. Clinical pregnancy is a complex process, and the influence of other factors on clinical pregnancy cannot be ignored.

Endometrial thickness is the most evaluated ultrasound ER indicator in previous studies. There was no difference between the pregnant and nonpregnant groups in endometrial thickness, which may be attributed to the fact that in our center, FET is performed only when the endometrial thickness is ≥8 mm. However, the CPR increased with endometrial thickening and was the highest when the endometrial thickness was 9–14 mm, which is consistent with previous research results, indicating that only moderate endometrial thickness is suitable for embryo implantation. This is likely because there was no difference in endometrial thickness between the two groups, and therefore, there was also no difference in the EV. However, when the EV was greater than 2 mL, the CPR increased significantly. Therefore, 2 mL was used as the threshold value in this study.

Although the pulsatility index (PI) and resistance index (RI) were considered effective indicators in the early years, subsequent studies reported that the uterine artery was not a direct endometrial blood-supply vessel and had limited value for ER evaluation (18, 32). Therefore, blood flow parameters such as the PI and RI of the uterine artery were not included in the present scoring system.

We constructed this comprehensive scoring system based on the limited predictive value of single ultrasound indices. There have been 6 previous studies on scoring systems, most of which showed good assessment ability, but most of the studies were early and retrospective (Table 3). The earliest scoring system was proposed in 1995 (20), and the measurement time was not specified. The scoring items included endometrial thickness, endometrial blood flow, endometrial layering, endometrial peristalsis, PI of uterine artery flow, myometrial echogenicity and myometrial blood flow. The second, proposed in 1998 (21), measured ER on Day 22 of the natural menstrual cycle before IVF. With advances in 3D technology, two articles published after 2020 (24) focused on the situation of the endometrium and its blood supply and omitted the observation of uterine artery and myometrial conditions. However, a paper published in 2002 (22) showed that the ultrasound scoring system was of limited value and could not better indicate the ER situation, which may be because the measurement in this article was performed on the HCG injection day and included many additional endometrial indicators, such as the myometrium echo, myometrium blood supply and uterine artery blood flow. This finding indicates that the parameters outside the endometrium may not be good indicators of ER. Which ultrasonic indicator to use, when to perform measurements and how to assign values may have a great impact on the prediction effect.

**Table 3:** Endometrial receptivity scoring system in previous studies and the present study.

Author	Studied population	Inclusion criteria	Measurement time	Included parameters	Total score range (clinical pregnancy rate)
Applebaum (20) (/, retrospective)	Infertile patients	Patients with no abnormalities in uterine shape or development, no other gross uterine abnormalities (e.g., significant masses) and a normal ovarian cycle without ovarian uterine dyscoordination, and without male infertility factor	Not mentioned	Endometrial thickness, endometrial layering, myometrial contractions, myometrial echogenicity, PI of uterine artery flow, endometrial blood flow, myometrial blood flow	0–20 (0–100%, significantly different)
Salle et al. (21) (*n* = 96, retrospective)	Infertile patients	The menstrual cycle of the month before IVF, women <38 years old	22^nd^ day of the menstrual cycle preceding IVF	Endometrial thickness, endometrial morphology, myometrial echogenicity, uterine artery PI, protodiastolic notch, end diastolic blood flow, endometrial blood flow	0–20 (0–42% with a score > 16, significantly different)
Baruffi et al. (22) (*n* = 562, prospective)	Infertile patients	Infertile patients submitted to ovarian stimulation for ICSI were studied on the day of administration of human chorionic gonadotropin	The day of hCG administration	Endometrial thickness, endometrial layering, myometrial contractions in 2 min, uterine artery doppler flow, endometrial power doppler, myometrial power Doppler, myometrial echogenicity	0–20 (12.0% with a score < 10–2–5.0% with a score of 18–20, did not differ significantly)
Khan et al. (23) (*n* = 200, retrospective)	Infertile patients	Primary infertility patients, age ranging from 24 to 43 years. The infertility duration ranged from 2 to 20 years, without associated male infertility, undergoing FET after the first stimulated IVF treatment	The 10th day of the FET menstrual cycle	Endometrial thickness, endometrial morphology, endometrial blood flow, myometrial echogenicity, uterine artery PI, end diastolic blood flow, myometrial blood flow	0–20 (0–9–7.4%, significantly different)
Jiao et al. (24) (*n* = 200, retrospective)	Ordinary patients	Patients who had not given birth but underwent artificial abortion 1–3 times (study group) and patients of childbearing age but who had not given birth and had no history of artificial abortion (control group)	The middle luteum phase (7–9 days after ovulation), in the AA group, ultrasonography was performed at 3 months after the last AA	Endometrial thickness, endometrial type, endometrial peristalsis (per min), EV, endometrial VFI	6–18 (AA group: 10.46 ± 2.99 vs. control group 13.49 ± 2.21, significantly different)
Zhang et al. (25) (*n* = 562, retrospective)	Infertile patients	Patients undergoing their first FET cycles. Patients with uterine anomalies, hydrosalpinx, endometriosis, PGT cycles, oocyte donation cycles, and nonhigh-quality embryos transferred were excluded	The morning on the transfer day of the FET cycle	Endometrial thickness, EV, echo of the functional layer of endometrium, endometrial central echogenic line, endometrial peristalsis, endometrial blood flow	0–12 (0–83.3% with a score of 8, significantly different)
This study (*n* = 197, prospective)	Infertile patients	Patients undergoing natural FET cycles after the first stimulated IVF treatment	The morning on the transfer day of the FET cycle	Endometrial morphology, endometrial blood flow, endometrial thickness, EV, endometrial FI, frequency of endometrial peristalsis	0–12 (0–100% with a score of 11, significantly different)

PI, pulsatility index; IVF, *in vitro* fertilization; ICSI, intracytoplasmic sperm injection; hCG, human chorionic gonadotropin; FET, frozen embryo transfer; AA, artificial abortion; EV, endometrial volume; VFI, vascularization flow index; PGT, preimplantation genetic testing; FI, flow index.

This was a prospective study, including the EV and FI as measured by 3D ultrasound. The constructed scoring system has good predictive ability, but there are still some limitations. First, the sample size, especially the verification sample size, was not large enough, the population involved was limited, and only natural cycles of FET were analyzed. The effectiveness of this scoring system for fresh embryos, modified frozen embryo cycles and natural pregnancy populations has yet to be verified in larger samples. In addition, although the scoring system has achieved certain evaluation effects, its AUC is not particularly ideal, which we speculate may be because the indicators currently included in the scoring system and their assigned values are not necessarily optimal. Perhaps with the advancement of ultrasound technology, new and more direct indicators will emerge, which will require further exploration with larger samples in the future. Third, at the time of writing, not all patients had been tracked to term, so this study only analyzed the relationship between ER and CPR, and further research is needed on the relationship between ER and live birth.

## Conclusion

A noninvasive ultrasound scoring system for ER has been proposed, and its evaluation value and ability have been verified. This system may provide a non-invasive guidance perspective, in conjunction with invasive assessments currently used in clinical practice, to achieve more effective embryo transfer. However, it should be noted that the limitations of this study should be taken into account when attempting to apply this scoring system of this study to clinical practice.

## Data availability statement

The original contributions presented in the study are included in the article/supplementary material, further inquiries can be directed to the corresponding author.

## Ethics statement

The studies involving humans were approved by the Ethics Committee of the Reproductive and Genetic Hospital of CITIC-Xiangya. The studies were conducted in accordance with the local legislation and institutional requirements. The participants provided their written informed consent to participate in this study. Written informed consent was obtained from the individual(s) for the publication of any potentially identifiable images or data included in this article.

## Author contributions

YO: Conceptualization, Formal analysis, Writing – original draft, Writing – review & editing. YP: Conceptualization, Formal analysis, Writing – original draft, Writing – review & editing. YM: Formal analysis, Resources, Writing – review & editing. MZ: Data curation, Resources, Writing – review & editing. FG: Data curation, Resources, Writing – review & editing. YL: Data curation, Resources, Writing – review & editing. XL: Conceptualization, Project administration, Resources, Supervision, Writing – review & editing.
