# Corrigendum: An endometrial receptivity scoring system evaluated by ultrasonography in patients undergoing frozen–thawed embryo transfer: a prospective cohort study

**DOI:** 10.3389/fmed.2024.1415825

**Published:** 2024-05-23

**Authors:** Yan Ouyang, Yangqin Peng, Yuyao Mao, Mingxiang Zheng, Fei Gong, Yuan Li, Xihong Li

**Affiliations:** ^1^Reproductive and Genetic Hospital of CITIC-Xiangya, Changsha, China; ^2^Clinical Research Centre for Reproduction and Genetics in Hunan Province, Changsha, China; ^3^School of Medicine, Hunan Normal University, Changsha, China; ^4^NHC Key Laboratory of Human Stem Cell and Reproductive Engineering, School of Basic Medical Sciences, Central South University, Changsha, China

**Keywords:** endometrial receptivity, three-dimensional ultrasound, ultrasonography, prospective, scoring system, noninvasive

In the published article, there was an error in affiliation 4. Instead of “Institute of Reproductive and Stem Cell Engineering, Central South University, Changsha, China,” it should be “NHC Key Laboratory of Human Stem Cell and Reproductive Engineering, School of Basic Medical Sciences, Central South University, Changsha, China.”

The authors apologize for this error and state that this does not change the scientific conclusions of the article in any way. The original article has been updated.

